# A Comprehensive Bioinformatics Analysis of the Nudix Superfamily in *Arabidopsis thaliana*

**DOI:** 10.1155/2009/820381

**Published:** 2009-07-02

**Authors:** D. Gunawardana, V. A. Likic, K. R. Gayler

**Affiliations:** ^1^Department of Biochemistry and Molecular Biology, Bio21 Molecular Science and Biotechnology Institute, University of Melbourne, Parkville, Victoria 3010, Australia; ^2^School of Biological Sciences, University of Sydney, New South Wales 2006, Australia

## Abstract

Nudix enzymes are a superfamily with a conserved common reaction mechanism that provides the capacity for the hydrolysis of a broad spectrum of metabolites. We used hidden Markov models based on Nudix sequences from the PFAM and PROSITE databases to identify Nudix hydrolases encoded by the *Arabidopsis* genome. 25 Nudix hydrolases were identified and classified into 11 individual families by pairwise sequence alignments. Intron phases were strikingly conserved in each family. Phylogenetic analysis showed that all multimember families formed monophyletic clusters. Conserved familial sequence motifs were identified with the MEME motif analysis algorithm. One motif (motif 4) was found in three diverse families. All proteins containing motif 4 demonstrated a degree of preference for substrates containing an ADP moiety. We conclude that HMM model-based genome scanning and MEME motif analysis, respectively, can significantly improve the identification and assignment of function of new members of this mechanistically-diverse protein superfamily.

## 1. Introduction

Nudix hydrolases are a diverse superfamily of pyrophosphatases found in all three domains—archaea, bacteria, and eukarya [1, 2]. More than 1800 open reading frames coding for Nudix hydrolases, in over 360 different species, have been identified by bioinformatics searches [3]. Members of the superfamily of Nudix hydrolases predominantly catalyze the hydrolysis of a wide range of small nucleotide substrates composed of a *nu* cleoside *di* phosphate linked to another moiety *X*, hence the name *Nudix* [4]. Nucleoside triphosphates (Npp-p), dinucleoside polyphosphates (Npp-p(n)N), nucleotide sugars (Npp-sugar), and mRNA (Npp-pRNA) are examples of such Nudix compounds. 

The Nudix hydrolases constitute a “mechanistically diverse superfamily”. This by definition is a group of evolutionarily related proteins that utilize a common mechanistic attribute, such as a specific partial reaction, intermediate, or transition state to catalyze different overall reactions with diverse substrate specificities [5]. In line with this definition, all members of the Nudix superfamily share a conserved amino acid sequence termed the Nudix box, the signature motif for Nudix hydrolases [1, 4]. It constitutes a key component of the catalytic site of these enzymes [4]. The Nudix motif, as defined by Bessman et al. [4], is comprised of 23 amino acids represented by the sequence G(X5)E(X7)REUXEEXGU, where U denotes a bulky hydrophobic amino acid, and X depicts any amino acid. Structural studies combined with site-directed mutagenesis have demonstrated that the Nudix box contains conserved residues essential for substrate hydrolysis [6–8]. Hydrolysis is mediated through nucleophilic substitution at *β*-phosphorus and is independent of the type of substrate cleaved [6–9]. Two glutamates, three residues apart, within an essential and conserved R**E**XXE**E** sequence motif within the Nudix box, play mandatory roles as the effector of catalysis and as a ligand to the enzyme bound metal ion cofactor, respectively [6–9]. 

In vivo studies have demonstrated the importance of individual Nudix enzymes in plants. Overexpression of gene At4g11980, encoding an ADP-ribose/ADP-glucose pyrophosphatase, reduced levels of both its substrate ADP-glucose and the biopolymer starch in *Arabidopsis* plants [10]. Inactivation of a fibroblast growth factor type Nudix enzyme (FGFTNE) gene (At4g12720) in *Arabidopsis* plants led to a pleiotropic phenotype with dwarfed growth, curled leaves, microscopic cell death, higher resistance to pathogens, and increased levels of reactive oxygen species (ROS) [11]. A T-DNA knockout of the same FGFTNE gene resulted in growth retardation, increased basal resistance to virulent *Hyaloperonospora parasitica*, and fourfold higher levels of the defense molecule salicylic acid, suggesting a deregulated defense mechanism [12]. T-DNA knockout of the Nudix gene At5g13570 coding for the *Arabidopsis* mRNA decapping enzyme similarly demonstrated its importance for plant growth and elongation [13]. Heterozygous mutation of At5g13570 resulted in stunted hypocotyls and roots, and the homozygous knockout was postembryonically lethal [13]. 

In spite of phenotypical evidence from gene inactivation studies and the characterization in vitro of selected plant Nudix hydrolases, understanding of the diversity and evolution of this enzyme superfamily in plants is incomplete. The most comprehensive bioinformatics analysis of the Nudix superfamily performed so far was restricted to three families of Nudix hydrolases and was based on sequence alignments alone [14]. With the exception of the human and yeast Nudix hydrolases [15], knowledge of the diversity of Nudix hydrolases in eukaryotic organisms, particularly plants, is limited. The first plant genome to be sequenced was that of *A. thaliana*, and genome resources on this organism are freely available for bioinformatics analyses [16]. We have undertaken an extensive analysis of the Nudix superfamily in *A. thaliana* to (1) assess the diversity of Nudix hydrolases in plants by gene identification and annotation, (2) classify families of enzymes within the Nudix superfamily based on sequence conservation, genetic architecture, and phylogenetic proximity, (3) identify motifs that could determine substrate specificities and functional classes of enzymes, and (4) analyze motifs to predict substrate specificities associated with particular motifs. It is hoped that a bioinformatics analysis incorporating sequence information, gene structures, evolutionary relationships, and motifs of putative functional significance will aid future genome/proteome studies of plant Nudix hydrolases, to identify Nudix genes and their encoded proteins from sequenced genomes and to facilitate the engineering of functional motifs for protein structure-function studies.

## 2. Materials and Methods

### 2.1. Hidden Markov Model Scans

The *Arabidopsis* proteome was scanned for putative Nudix hydrolases in three analytical steps. Initially the conceptual proteome of *A. thaliana* was scanned with hidden Markov models (HMMs) [17] based on the “seed” and “full” models for the superfamily of Nudix hydrolases from the PFAM database. The total non-redundant sequences from both the “seed” and “full” model based searches were combined. A second analysis of the *Arabidopsis* conceptual proteome was performed using HMM models derived from the two-element fingerprint (two consensus sequence motifs of 15 and 16 residues) that provides a signature for the Nudix box in the PROSITE database. In all HMM scans the cutoff *E*-value was 0.01. The total entries that matched the search criteria from the two searches (using the PFAM and PROSITE sequences) were pooled. The non-redundant pooled sequences were further reduced to those containing the hexaresidue motif REXXEE, a region conserved and essential within the active site of Nudix hydrolases. Finally, sequences not containing the 23 residue Nudix box consensus G(X5)E(X7)REUXEEXGU were discarded. This final selection was by individual manual scrutiny.

### 2.2. Multiple Sequence Alignment of Protein and DNA Sequences

Multiple protein sequences were aligned with the European Bioinformatics Institute (EBI) CLUSTALW server and the CLUSTALW algorithm [18] implemented within Data Analysis in Molecular Biology and Evolution 4.13 (DAMBE) [19]. For the alignment of DNA sequences, the corresponding protein sequences were first aligned using the CLUSTALW algorithm and subsequently converted to the respective DNA sequences.

### 2.3. Determination of Genetic Architecture and Intron Phases

Intron-exon architecture and intron phases for each Nudix hydrolase gene were obtained from the *Arabidopsis* Information Resource (TAIR) [20] and the Xpro database [21].

### 2.4. Phylogenetic Analysis

Maximum parsimony trees for the *Arabidopsis* Nudix hydrolases were created using DAMBE version 4.13 [19]. The relationships between adjacent nodes were based on bootstrap support from 500 replicates. The gene trees were created either as unrooted trees or considering the gene At1g68760 as the outgroup. The gene product of At1g68760 catalyzes an essential step in the folate synthesis pathway, only fully conserved in plants and bacteria [22].

### 2.5. Motif Analysis

The program MEME version 3.0.4 was used for the elucidation of motifs in Nudix sequences [23]. MEME was run locally with the parameters set to their default values. The model “zoops” was used, and the motif widths were constrained to between 6 and 90 residues.

### 2.6. Homology Modeling

Homology modeling of the three-dimensional structures of proteins was performed using SWISS-MODEL [24], and the structures further analyzed using PyMOL version 0.97.

### 2.7. Assessment of Gene Duplication Events

The Nudix sequences from five genomes (*Synechococcuselongatus*, *Saccharomycescerevisiae*, *Homosapiens*, *Oryzasativa*, and *A*. *thaliana*) comprising of a broad taxonomical spectrum were used to create a gene tree. The genomes were representative of cyanobacteria, unicellular eukaryotes, mammals, and monocotyledonous and dicotyledonous plants. The protein sequences of the Nudix hydrolases from the above organisms were first downloaded from the PFAM database, then aligned using the CLUSTAL W algorithm [18], and finally reverse translated into the corresponding DNA sequences. The gene tree was created by maximum parsimony analysis using DAMBE version 4.13 [19]. Adjacent nodes were derived from bootstrap support from 250 replicates. The binary species tree for the corresponding organisms was downloaded from the NCBI taxonomy server. The gene trees were reconciled to the species tree using NOTUNG 2.0 beta using the default parameters [25].

## 3. Results

### 3.1. Identification of Nudix Hydrolases in *A. thaliana*

**Figure 1:**
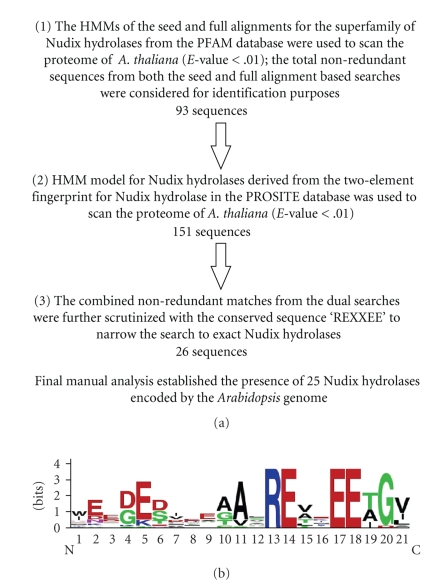
Identification of Nudix hydrolases encoded by the *Arabidopsis* genome. (a) Flowchart by which putative Nudix proteins encoded by the *Arabidopsis* genome were identified within the PFAM and PROSITE databases. (b) Consensus for the final 21 residues of the Nudix motif within the identified members of the Nudix superfamily in *A. thaliana*.

The identification of all members of the superfamily of Nudix hydrolases in *A*. *thaliana * was sought using a hidden Markov model-(HMM-) based approach to detect the widest possible array of related sequences including many potential false positives (Figure 1(a)). The scanning of the *Arabidopsis* proteome for these putative Nudix hydrolases was first conducted using the sequence resources of the PFAM database [26]. The HMMs based on “seed” and “full” alignments for the superfamily of Nudix hydrolases from the PFAM database were used to scan the *Arabidopsis* proteome with the *E*-value cutoff of 0.01. The *E*-value cutoff of 0.01 is arbitrary, but a typical one used in BLAST and HMM searches. There were 61 hits for the “seed”, and 74 hits for the “full” model. The intersection of two sets of sequences (a non-redundant set) contained 93 sequences and was taken as the first set of putative Nudix hydrolases (Figure 1(a)). 

In a second step, HMMs for the Nudix superfamily were built with the aid of the signature motifs characteristic of Nudix hydrolases within the PROSITE database [27, 28]. In PROSITE, the Nudix hydrolases are represented by a two-element fingerprint (two consensus motifs of 15 and 16 residues) that provides a signature for the Nudix hydrolase superfamily (PROSITE accession code PS00502, Nudix hydrolase family motifs II-4 and I-4). The two motifs span the full length of the Nudix (MutT) domain and include the region encoded by the PROSITE pattern MUTT (PS00893). The motifs (I and II) which form the two-element fingerprint can be described by the consensus sequences “(X5)G(X5)E(X3)” and “(X5)REUXEEXGU(X2)”, respectively, where U denotes a hydrophobic residue, and X specifies any residue, with an overlap between the final residue of motif I and first residue of motif II. The HMMs built from the two PROSITE motifs were used to scan the *A. thaliana* conceptual proteome to identify putative Nudix enzymes. There were a total of 151 unique hits matching the two HMMs with the *E*-value <.01 (Figure 1(a)). 

The entries that matched the search criteria using the PFAM-based search (93 sequences) and PROSITE-based search (151 sequences) were pooled, and the non-redundant sequences (intersection of the two sets, a total of 103 sequences) retained for further analysis. These sequences were further scrutinized for the presence of the REXXEE motif, a region conserved within the active site of Nudix hydrolases [15]. This hexaresidue consensus contains two essential glutamates (R**E**XXE**E**) that are central components of the catalytic mechanism—the first acts as the effector of catalysis that deprotonates the attacking water molecule, whereas the second provides a ligand to the enzyme-bound metal ion cofactor [9]. This eliminated all but 26 sequences (Figure 1(a)). 

Further manual analysis for the presence of the complete 23 residue Nudix box signature sequences (G(X5)E(X7)REUXEEXGU) reduced the total number to 25 Nudix hydrolases. Figure 1(b) shows the extent of conservation of these residues amongst these 25 Nudix hydrolases. As demonstrated in Figure 1(b), in spite of the essential conservation of the REXXEE sequence, subtle variations to the 23 residue consensus were present in identified members of the Nudix superfamily, mainly in residues not directly involved in the mechanism of catalysis.

For this study, the Nudix superfamily was defined by and limited to genes encoding a complete 23 residue signature sequence. Nevertheless the search for Nudix hydrolases within the *Arabidopsis* genome was extended to check for GDP-mannose mannosyl hydrolases, a Nudix family where the REXXEE signature sequence is absent. Direct BLASTP searches were carried out using the *E. coli* GDP-mannose mannosyl hydrolase against the *Arabidopsis* proteome. However, not a single putative GDP-mannose mannosyl hydrolase was detected. The absence of GDP-mannose mannosyl hydrolases in *A. thaliana* was consistent with the restriction of this family entirely to gram-negative bacteria. 

As a separate measure of verification, the 23 residue Nudix box sequence patterns were utilized to search the NCBI *A. thaliana* database. The patterns shown in Table 1 were used to search the database using the Seedtop program from NCBI. 

The total complement of Nudix hydrolase sequences (25) identified using HMMs were not found within the 62 and 107 sequences identified by pattern matching. The products of genes At1g28960, At2g04430, At4g11980, and At5g45940 were not present in both sequence listings (data not shown) demonstrating the limitation of this method to identify the total compliment of Nudix hydrolases encoded by the *Arabidopsis* genome. Therefore, we conclude that an organized HMM-based analysis, as employed in this study, is a better alternative to pattern matching for the identification of members of this enzyme superfamily.

### 3.2. Classification and Annotation of Families of Enzymes within the Nudix Superfamily

Nudix hydrolases constitute a superfamily, as defined in the Structural Classification of Proteins Database (SCOP) where proteins with low sequence identity but whose structures and functional features suggest a common evolutionary origin are classified as a superfamily [29]. In this context, Nudix hydrolases possess a common fold (*α* + *β* fold) and a conserved mechanism of catalysis (substitution at *β*-phosphorus) but relatively low overall sequence similarity to each other. By contrast, the term “family” as defined by SCOP is restricted to a group of proteins that have both a common evolutionary origin and residue identities of 30% or greater [29]. Therefore, the scores of pairwise alignments were used as the foundation for the classification of families within the Nudix superfamily with a threshold score of 30 assigned as the cutoff value. Pairwise alignment scores were calculated as the number of identities in the best alignment divided by the number of residues compared, excluding gap positions and presented as percent identity scores. 

Table 2 lists the families where sequence identity was >30%. The full matrix of pairwise sequence alignment scores is presented as in Supplementary Table A in Supplementary Material available online at doi: 10.1155/2009/820381. The nomenclature used in this paper is derived from gene identities as annotated in the TAIR database and not their protein counterparts, since for five of the Nudix hydrolases (At1g30110, At3g10620, At5g06340, At5g13570, At4g25440) no identity has been presented using the convention for nomenclature of *Arabidpopsis* Nudix proteins (Prefix-AtNUDT). For consistency purposes, the Nudix genes/gene products from *A. thaliana* will be identified in the standard format for chromosome-based nomenclature for *Arabidopsis* genes as described in the TAIR database throughout this publication. 

Six proteins failed to align at a level >30% to any other protein identified from HMM-based proteome scanning (Supplementary Table A) and were designated as those represented by a single family member. The functional classification of each family, as listed in Table 2, was based on similarities to enzymes of known substrate preferences from other species. BLASTP searches were carried out in this study on each of the gene products listed in Table 2, and a >30% sequence identity level to enzymes of known function was used as the basis for their annotation. The annotations assigned in this way agreed with those in the NCBI database.

In all, nine protein families were assigned functions, and there were two further families of unknown function. The nine designated families were Ap_*n*_A hydrolases, diphosphoinositol polyphosphate phosphohydrolases (DIPPs), FGFTNEs, coenzyme A pyrophosphatases, NADH hydrolases, dihydroneopterin triphosphate phosphohydrolases (DHNTPs), ADP-ribose pyrophosphatases, isopentenyl diphosphate isomerases, and mRNA decapping enzymes (Table 2). The designation of FGFTNEs was based on similarity of these proteins to a protein fragment encoded by the human fibroblast growth factor mRNA. In this study, this family has been designated as FGFTNEs, due to both the similarity of these proteins to type 2 fibroblast growth factors and the clear presence of the signature Nudix motif in all familial members. 

**Figure 2:**
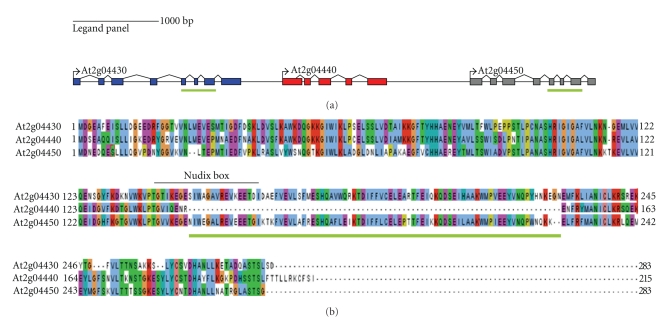
Architecture of a putative FGFTNE pseudogene. (a) Intron-exon architecture of the At2g04430, At2g04440, and At2g04450 genes. (b) The sequence alignment of the gene products of At2g04430, At2g04440, and At2g04450. The exons that are missing in the At2g04440 gene and the analogous protein sequence are underlined in green. The Nudix box region is also marked.

One pseudogene, with high sequence identity to the FGFTNEs, was also identified in the preliminary HMM-based scanning of the *Arabidopsis* proteome. This pseudogene which is annotated as At2g04440 is found between two putative Nudix genes At2g04430 and At2g04450, which are located within a short stretch of 6 kb of the genome (Figure 2). Genes At2g04430 and At2g04450 are both putative FGFTNEs with 58% sequence identity to each other at the protein level. The encoded protein of the pseudogene At2g0440 also shows significant overall similarity to all other FGFTNEs. However the At2g04440 gene product failed to appear amongst the 25 Nudix hydrolases determined by the HMM-based scanning of the *Arabidopsis* proteome, due to the absence of a region of 84 residues inclusive of the Nudix box and hence the catalytic site (Figure 2). It is likely that the *Arabidopsis* genome contains other pseudogenes which were not identified in this study due to their more extensive sequence divergences. By contrast, it is unlikely that any of 25 Nudix genes identified in this study are pseudogenes. Evidence for expression of the genes as mRNAs was sought by PCR-based screening of an *Arabidopsis* cDNA library created from mRNA of 42 day old *A. thaliana* Col-0 ecotype plants. 14 of the 25 Nudix genes identified in this study were tested. All 14 were detected as cDNAs in the *Arabidopsis* cDNA library (Table 2) confirming that all 14 genes were capable of expression at the mRNA level. The expression level data were limited to the above genes since the cDNAs of these 14 genes were used for their initial cloning in to maintenance/expression vectors and for the production of selective proteins in bacterial expression systems, for subsequent enzymological studies. The characterization of one of the proteins, the mRNA decapping enzyme (gene product At5g13570), has already been published in the journal *NucleicAcids* *Research* [30].

### 3.3. Analysis of Intron-Exon Architecture

Genes of each of the families of the Nudix superfamily showed a high degree of conservation of intron-exon architecture. Introns have been mapped and characterized in most genomes of model organisms including *A. thaliana*. Spliceosomal introns have been classified based on the position of the intron with respect to the reading frame of the gene at the intron-exon boundaries [31]. Phase 0 introns fall between two codons whereas phase 1 and phase 2 introns fall at the termination of the first and the second base, respectively, of the final codon in the 5′ exon [31]. Intron phases are likely to be conserved since the modification of a frame at the proximal side of an intron would necessitate a retaliatory change at the distal exon to maintain the reading frame [31, 32]. In our study, the intron-exon structures of the Nudix hydrolases were obtained from the Xpro and the TAIR databases [20, 21]. A high degree of conservation of intron phases was detected in multimember families of the Nudix hydrolases (Table 3) as an indicator of extensive conservation of intron-exon architecture within these families. Such conservation of intron phases in families with multiple members is indicative of the above evolutionary difficulty in inducing frame-shifts within the genetic boundaries of a single gene. As further shown in Table 3, there are striking distinctions in the arrangement of introns and intron phases between families of the Nudix superfamily. It was concluded, based on the preservation of arrangement of intron phases within families and the striking dissimilarity of intron phase patterns between families, that there is a strong genetic basis for the subgrouping into families otherwise assigned by pairwise alignment.

### 3.4. Phylogenetic Analysis

**Figure 3:**
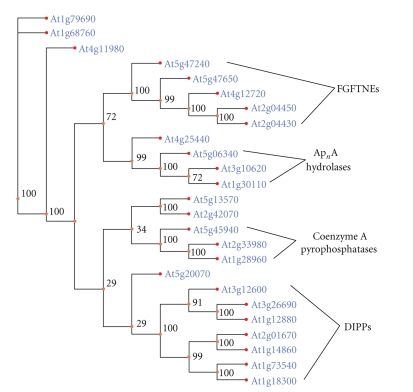
Maximum parsimony tree depicting the evolutionary relationships of the identified Nudix hydrolase genes in *A. thaliana.* Representative bootstrap values are shown as a percentage from 500 bootstrap replicates. The tree was rooted using the At1g68760 gene.

Multiple sequence alignment of the 25 Nudix hydrolases was used as an entry for a phylogenetic analysis using DAMBE software. Protein level alignments were created using the CLUSTALW algorithm and converted to the corresponding DNA sequences for phylogenetic analysis. Gene trees were created based on the maximum parsimony method with bootstrap support from 500 replicates (Figure 3). 

The outgroup used to root the gene trees was a compromise selected after assessing the occurrence of each of the 25 sequences between species seeking ancestral genes that were not prevalent in most eukaryotic lineages. Gene At1g68760 was used for tree rooting purposes, even though it cannot be considered a superlative outgroup, in terms of distinctness and ancestry. Even though homologues of this gene could be found in other eukaryotes, it was chosen as the outgroup due to its unique position as essential for plants but not for animals. It encodes a dihydroneopterin triphosphate hydrolase, an essential enzyme in the folate synthesis pathway in bacteria and plants [33]. Humans and other mammals lack a complete folate synthesis pathway and are dependent on folate from plant and bacterial sources [22]. In addition, the At1g68760 protein is the smallest member of the Nudix superfamily in *Arabidopsis thaliana* consisting of 147 amino acids. Due to the dependence of exon integration at the 5′ and 3′ end of proteins for evolutionary expansion in function, the At1g68760 protein serves as the smallest possible “core unit” to base the phylogenetic analysis. 

The gene tree created using the maximum parsimony method shows the phylogenetic proximity of multimember families with the DIPPs, FGFTNEs, coenzyme A pyrophosphatases, and Ap_*n*_A hydrolases forming monophyletic clusters (Figure 3). Further, there was high bootstrap support at nodes between members of a single family (Figure 3). Overall, the phylogenetic analysis was consistent with the classification of families obtained using pairwise alignments and intron phase analysis.

### 3.5. Motif Analysis

**Figure 4:**
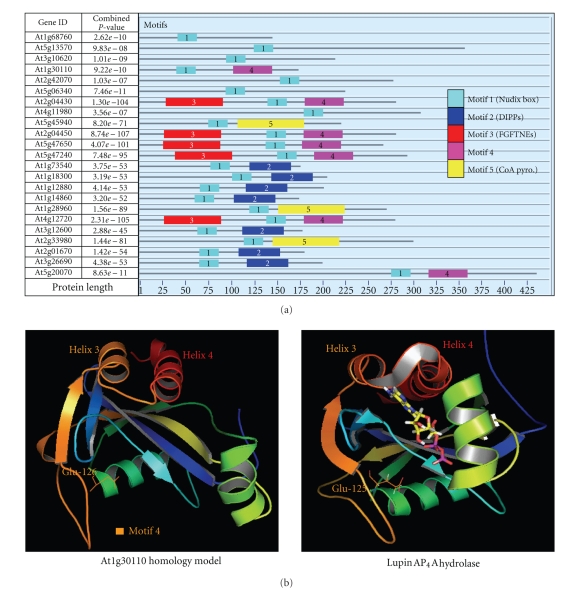
Conserved motifs in *Arabidopsis* Nudix hydrolases. (a) Different colored boxes and their designated numbers indicate separate and distinct motifs of 23 of the 25 Nudix hydrolases identified using MEME [23]. The At4g25440 and At1g79690 gene products were omitted from the figure for convenience of presentation. (b) Comparison of the homology model of the At1g30110 protein with that of the structure of the lupin Ap_4_A hydrolase. The structure of the At1g30110 gene product (Left), a putative Ap_*n*_A hydrolase from *A. thaliana * modeled against the known structure of the lupin Ap_4_A hydrolase (PDB ID-1JKN) in a complex with ATP-MgF_x_ (Right). Motif 4 is colored orange in the homology model, and helices 3 and 4 are marked. The loop region which contains the metal-binding Glu-125 in the lupin enzyme structure, the equivalent loop in the At1g30110 homology model, and the side chain of analogous Glu-126 are also depicted. The catalytic helix containing part of the conserved Nudix box is shown in green in each model.

Conserved motifs in protein sequences that are indicative of functional sites and are conserved between functionally similar proteins [26] were sought within the Nudix superfamily. The conserved motifs detected in the Nudix hydrolases by the program MEME [23] are shown in Figure 4(a). Motif 1 in Figure 4(a) is the Nudix box, and by definition all 25 sequences of the Nudix superfamily in *A. thaliana* contained this motif. 

As demonstrated in Figure 4(a), four other conserved motifs were found outside of the Nudix box in various members of the superfamily of Nudix hydrolases. Three of the identified motifs were unique to the DIPP (motif 2), FGFTNE (motif 3), and coenzyme A pyrophosphatase (motif 5) families, respectively. Such motifs are putative candidates for structural domains involved in positioning of the primary substrate of these families. Surprisingly, no motif unique to the fourth multimember family, the Ap_*n*_A hydrolases, was detected in our analysis. Rather another motif (motif 4), which is present in all members of the FGFTNE family, was also found in the products of genes At1g30110 and At5g20070, belonging to the Ap_*n*_A hydrolase and NADH hydrolase families, respectively. 

We have studied motif 4 in more detail. This motif is located in a region significant for substrate binding, at least in the At1g30110 gene product, as shown by homology modeling of the At1g30110 gene product with the structure of the lupin Ap_4_A hydrolase (Figure 4(b)). The three-dimensional structure of the Ap_4_A hydrolase from *Lupinus angustifolius* was used in this comparison as the nearest such structure available from a plant enzyme [34]. Homology modeling positioned motif 4 in a region analogous to the highly mobile helix-loop-helix (helix 3-loop-helix 4), the preceding beta sheet, and a loop region that contained a metal-binding glutamate (Glu-125) in the lupin enzyme (Figure 4(b)). This highly mobile region was shown to be central to the binding of the nucleotide substrates in the lupin enzyme [35]. Helix 3, helix 4, and the linking loop region moved outward to accommodate the ATP-MgF_x_ complex at the substrate binding site [35]. The backbone and side chains of amino acids within the intervening loop region between helices 3 and 4 interacted with the adenine moiety of the ATP-MgF_x_ complex [35]. It is concluded that motif 4 is a potential substrate binding site that has evolved to accommodate different but perhaps related substrates in a range of Nudix families. 

Though well separated in linear sequence from the Nudix box, motif 4 and the corresponding region in the lupin Ap_4_A hydrolase are both placed in close proximity to the conserved catalytic helix within the Nudix motif in the 3D models (Figure 4(b)). Motif 4 in fact contains Glu-126 equivalent to the catalytically-essential glutamate Glu-125 in the lupin enzyme that contributes a ligand to an obligatory divalent cation at the active site [34]. This further emphasizes the potential of motif 4 to affect substrate hydrolysis.

### 3.6. Divergent Enzymes Containing Motif 4

Two enzymes annotated as hydrolases of Ap_4_A (At1g30110) and NADH (At5g20070) and all members of the FGFTNE family contain motif 4. We performed bioinformatics analyses on the protein sequences containing motif 4 to investigate the putative functions of this promiscuous motif. 

**Figure 5:**
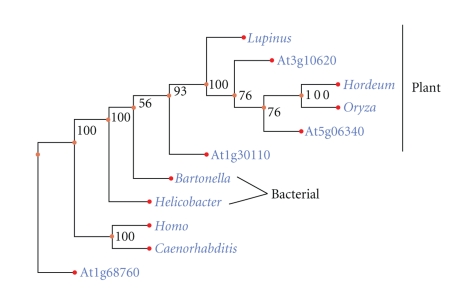
Phylogenetic analysis of Nudix Ap_*n*_A Hydrolases. Maximum parsimony tree depicting the evolutionary relationships of plant (*A. thaliana*, *L. angustifolius*, *O. sativa*, *H. vulgare*), animal (*Homo sapiens, Caenorhabditis elegans*), and bacterial (*Helicobacter pylori, Bartonella bacilliformis*) Ap_*n*_A hydrolase genes. Representative bootstrap values are shown as a percentage from 500 bootstrap replicates. The tree was rooted using the At1g68760 gene encoding a dihydroneopterin triphosphate hydrolase from *A. thaliana*

**Figure 6:**
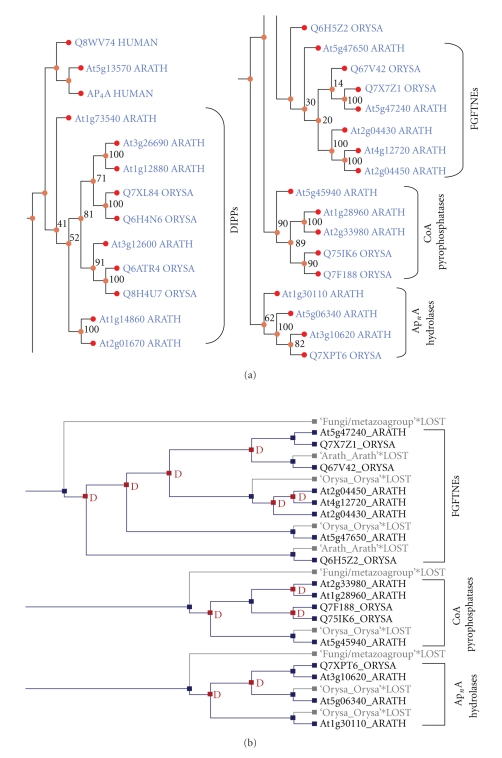
Gene duplication events in selective Nudix families. Maximum parsimony tree (selective regions shown adjacent to each other) depicting the phylogenetic relationships of Nudix hydrolase genes in the species *A. thaliana*, *O*. *sativa*, *H*. *sapiens*, *S*. *cerevisiae*, and *S*. *elongatus* rooted using the *NudC* gene from the gram negative bacterium *Vibrio cholerae.* Representative bootstrap values are shown as a percentage from 250 bootstrap replicates. ARATH and ORYSA are standard terminology for *A. thaliana * and *O*. *sativa*, respectively, according to the UniProt knowledgebase [37]. (b) Gene duplication events of the Ap_*n*_A hydrolase, coenzyme A pyrophosphatase, and FGFTNE families of Nudix hydrolases in *A. thaliana*. The gene tree from the species *A. thaliana*, *O*. *sativa*, *H*. *sapiens*, *S*. *cerevisiae*, and *S*. *elongatus* (Figure 6(a)) was reconciled with the corresponding binary species tree from NCBI as described in materials and methods section. ▪ D indicates a duplication event.

Bioinformatics analyses of the protein sequences of the At1g30110 and At5g20070 genes demonstrated distinctive features in both proteins. The At1g30110 gene product in particular appears to be quite distinct from all other plant Ap_*n*_A hydrolases. Sequence alignment revealed differences that distinguished At1g30110 from other plant Nudix Ap_4_A hydrolases, those from *A. thaliana*, *Lupinus angustifolius*, *Hordeum vulgare*, and *Oryza sativa*, none of which have a recognizable motif 4 (Supplementary—Figure A). Further, the At1g30110 gene product lacks a large N-terminal extension, a likely preprotein region, found in all other plant Ap_*n*_A hydrolases (Supplementary—Figure A). The inability to identify a marker motif common to all members of the Ap_*n*_A hydrolases in *A. thaliana* from MEME analysis, is further evidenced that there is significant divergence of the At1g30110 gene product away from its *Arabidopsis* counterparts. Phylogenetic analysis of ten plant, bacterial, and animal Nudix Ap_*n*_A hydrolases demonstrated that the At1g30110 gene product holds a unique evolutionary niche: outside of the cluster of other plant Nudix hydrolases and close to the bacterial Ap_*n*_A hydrolases (Figure 5). Analysis of gene duplication events also suggested that At1g30110 originated prior to the division of the dicotyledons (*A. thaliana*) and monocotyledons (*Oryza sativa*) lineages (Figure 6). Gene At1g30110 is likely to be an ancestral state of plant Nudix hydrolases from which the remaining Ap_*n*_A hydrolase genes have undergone some degree of divergent evolution. Recent biochemical studies have demonstrated that the At1g30110 gene product hydrolyzes Ap_4_A as the preferred substrate in the presence of Mn^2+^ ions [36]. 

The 438 amino acid At5g20070 gene product contains the signature sequence SQPWPFP_S, found in all members of the NADH hydrolase family [14], and has been demonstrated to be active on both NADH and NADPH [38]. However, it has been shown that unlike other Nudix NADH hydrolases, which are localized to peroxisomes, the *Arabidopsis* counterpart is targeted to chloroplasts [38]. 

All members of the FGFTNE family, although resembling type 2 fibroblast growth factors, are involved in the hydrolysis of both ADP-ribose and NADH [39]. Fibroblast growth factors are regulatory peptides that are often secreted and belong to a separate protein superfamily [40]. The functional basis for the sequence similarity between the FGFTNEs and fibroblast growth factors is yet unknown. It is likely that signaling functions are associated with this Nudix family, and evidence from in vivo studies have already demonstrated that pleiotropic phenotypes result from the inactivation of a single FGFTNE gene [11, 12]. In spite of several potential roles for FGFTNEs, their enzymatic capabilities to hydrolyze both NADH and ADP-ribose are of significance for the regulation of these molecules. The presence of motif 4 in all members of the FGFTNE family points to a role for this motif, in the hydrolysis of substrates containing an ADP moiety. Therefore, it appears that all proteins containing motif 4 are active on substrates such as NADH, ADP-ribose, and Ap_4_A that contain a terminal ADP moiety.

## 4. Discussion

In this study, 25 Nudix hydrolases encoded by the genome of *Arabidopsis thaliana* were identified by the combination of hidden Markov model searches and manual refinement of search results. A similar attempt by Ogawa et al. [39] to identify the total complement of Nudix hydrolases in *A. thaliana* using the NCBI database did not include four of the sequences identified in this study. The same four enzymes were absent in the study by Bartsch et al. [12]. Neither the three members of the Ap_*n*_A hydrolase family (At1g30110, At3g10620, At5g06340) nor the mRNA decapping enzyme (At5g13570) was identified in the studies by Ogawa et al. [39] and Bartsch et al. [12]. Further, three of the enzymes identified as Nudix hydrolases in Ogawa et al. [39] did not contain the conserved REXXEE sequence within the Nudix box. By contrast, the 25 Nudix hydrolases identified in our study contained the hexaresidue motif within the Nudix box sequence. 

In a separate study by Muñoz et al. [10], 31 Nudix hydrolases were identified by database searches. However, six of the identified proteins did not fit the criteria for classical Nudix hydrolases, and unless biochemical evidence is presented to back up the claims, they cannot be annotated as Nudix enzymes. The debated Nudix enzymes are (1) gene product of At2g04440, which we have demonstrated earlier to be a product of a pseudogene; (2) At3g02780 and At5g16440 gene products, which are isopentenyl diphosphate isomerases lacking Nudix motifs; (3) gene products of At5g19460 and At19470, both of which lack the essentially conserved REXXEE hexaresidue sequence within the Nudix box. Ogawa et al. [38] demonstrated that gene products of At5g19460 and At19470 are incapable of hydrolyzing any of the classical Nudix substrates, with the exception of the diphosphoinositol polyphosphates, which were not tested in this study, further validating the claim that they fall outside of the Nudix superfamily; (4) gene product of At3g46200, which again lacks the essentially conserved REXXEE sequence, has not been demonstrated to be active on any Nudix substrate [39].

In summary, our survey confirms that, like most eukaryotic genomes, the *Arabidopsis* genome codes for multiple Nudix enzymes. Unicellular yeast encodes six Nudix hydrolases and the number of functional Nudix hydrolases in humans is at least 24 [15]. Furthermore, this study clearly demonstrates the power of HMMs to identify accurately all representative members of a mechanistically diverse protein superfamily and to minimize misannotations that can arise from manual database searches. 

The identified Nudix hydrolases were classified into families by pairwise sequence alignments and the classification substantiated first using conserved genetic features such as intron phases and subsequently by assessing the phylogenetic proximity of individual family members. Of the identified families, the DIPPs and FGFTNEs in particular have expanded in *A. thaliana* compared to the representative members of these families in humans where four DIPPs and one FGFTNE have been reported [15]. Members of multimember families retained similar intron phases and were of a monophyletic origin, clearly demonstrating that the classification of families based on a 30% sequence identity level was accurate in assigning members to particular families. Whether the same genetic architecture can be extended to include other plant and animal Nudix genes remain to be seen. Interspecies preservation of intronic phases within gene families is less likely since increases in gene number can be traced back to either whole genome duplication events or segmental gene duplications arising from a single genome. For example, from searching the data of the study by Blanc et al. [41], the increased presence of DIPPs in *Arabidopsis thaliana* was determined to have originated from recent segmental duplication events (Table 4). 

According to the prevailing theory of enzyme evolution suggested by Petsko et al. [42], a majority of “new” substrate specificities are added on to the existing chemistry of catalysis by sequence divergences within substrate binding sites. Accordingly, the families of enzymes containing the catalytic Nudix motif in *A. thaliana* appear to have evolved to accommodate broad but familial substrate specificities as well as overlapping interfamilial substrate preferences, using modifications to their substrate binding sites. Computational motif analysis identified 4 motifs in Nudix enzymes in addition to the Nudix box that was by definition conserved between all identified members. Three of these were restricted to and present in all members of particular families. We conclude that motifs 2, 3, and 5 are markers of the DIPP, FGFTNE, and coenzyme A pyrophosphatase families, respectively. 

One motif, motif 4, was found in enzymes with annotated activities to NADH and Ap_4_A substrates and in FGFTNEs. The product of the At1g30110 gene hydrolyzed Ap_4_A, and the At5g20070 gene product hydrolyzed NADH, whereas all members of the FGFTNE family were capable of hydrolyzing both ADP-ribose and NADH [36, 38, 39]. An ADP moiety is common to each of these substrates. Motif 4 in the At1g30110 gene product was mapped onto the substrate binding region suggesting that motif 4 directly or indirectly aids the binding of ADP-containing substrates to this region of the protein. Motif 4 appears to be a unique event of convergent evolution within the Nudix superfamily, where substrate binding sites of divergent proteins have evolved analogous motifs to accommodate overlapping or related substrate specificities.

## 5. Conclusions

We have demonstrated that HMM model-based genome scanning and MEME motif analysis have significantly improved the accuracy of identification and annotation of Nudix hydrolases encoded by the *Arabidopsis* genome. We conclude that deciphering the diversity, organization, and phylogeny of Nudix genes should facilitate future annotation of Nudix genes within sequenced genomes in other organisms. The utilization of intron-phases appears particularly relevant in assessing the evolution of gene families within this enzyme superfamily. Identification of motifs specific for three families and a fourth motif associated with particular substrate preferences suggests that similar analyses should aid in the assignment of function of new members of this mechanistically diverse protein superfamily in other organisms. Identification of the location of motif 4 adjacent to the catalytic site within the 3D structure of the Nudix enzyme has focused attention on this region of the protein as one undergoing evolution to allow access to differing substrates. In doing so, it highlights the potential importance of not only motif 4 but also the other family specific motifs 2, 3, and 5 as regions of the enzymes upon which to focus future structure-based studies and protein engineering efforts to understand the evolution of enzymatic activities.

## Supplementary Material

Supplement Table A presenrs:
Matrix of CLUSTALW scores from pairwise sequence alignment. Boxes where >30% sequence identity was detected by CLUSTALW alignment are colored in orange.
Supplement Figure A presenrs:
Sequence alignment of ApnA hydrolases from plants.
The protein products of the *Arabidopsis* genes At1g30110, At3g10620 and At5g06340 and their counterparts from *Lupinus angustifolius* (gi:1888557), *Oryza sativa* (gi:50929793) and *Hordeum vulgare* (gi:2564253) are aligned using the CLUSTAL W algorithm. Motif 4 present only in the At1g30110 gene product is underlined in red. The N-terminal extensions forming putative preprotein sequences in all proteins excluding the At1g30110 gene product are underlined in green.

## Figures and Tables

**Table 1:** 

Pattern	*A. thaliana* matches
G-x(5)-E-x(7)-R-E-x(2)-E-E-x-G-x	107
G-x(5)-E-x(7)-R-E-[VILMFWC]-x-E-E-x-G-[VILMFWC]	62

**Table 2:** The putative Nudix hydrolases identified by the HMM based approach. The locus ID, the designated protein and their assigned families are shown.

Gene ID	Nomenclature of protein (NCBI)	Family
At1g30110*	—	Ap_*n*_A hydrolase
At3g10620*	—	Ap_*n*_A hydrolase
At5g06340*	—	Ap_*n*_A hydrolase
At1g12880*	AtNUDT12	DIPP
At1g14860	AtNUDT18	DIPP
At1g73540*	AtNUDT21	DIPP
At1g18300*	AtNUDT4	DIPP
At2g01670	AtNUDT17	DIPP
At3g12600*	AtNUDT16	DIPP
At3g26690	AtNUDT13	DIPP
At1g28960	AtNUDT15	Coenzyme A pyrophosphatase
At2g33980	AtNUDT22	Coenzyme A pyrophosphatase
At5g45940	AtNUDT11	Coenzyme A pyrophosphatase
At2g04430	AtNUDT5	FGFTNE
At2g04450*	AtNUDT6	FGFTNE
At4g12720*	AtNUDT7	FGFTNE
At5g47240*	AtNUDT8	FGFTNE
At5g47650	AtNUDT2	FGFTNE
At1g68760*	AtNUDT1	Dihydroneopterin triphosphate hydrolase
At1g79690*	AtNUDT3	Isopentenyl diphosphate isomerase
At2g42070	AtNUDT24	Unknown
At4g11980	AtNUDT14	ADP-ribose pyrophosphatase
At5g13570*	—	mRNA decapping enzyme
At5g20070*	AtNUDT19	NADH hydrolase
At4g25440	—	Unknown

*Nudix genes amplified by PCR from the Arabidopsis cDNA library.

**Table 3:** The distribution of exons and intron phases in multimember families of Nudix hydrolases in *A. thaliana* emphasizing the conservation in intron/exon architecture and of intron phase patterns within families.

Family	Members	Number of exons/size of protein modules encoded by the exons (N → C)	Intron phases N → C
Ap_*n*_A hydrolases	At1g30110	5–22,17,31,31,74	0,0,0,2
	At3g10620	6–77,17,31,31,39,21	0,0,0,2,0
	At5g06340	6–76,17,31,31,39,33	0,0,0,2,0

DIPPs	At1g18300	2–63,144	2
	At1g73540	2–43,134	2
	At1g14860	4–23,36,28,89	2,0,0
	At3g12600	4–23,39,28,90	2,0,0
	At1g12880	5–23,42,28,50,53	2,0,0,0
	At2g01670	5–28,35,28,43,48	2,0,0,0
	At3g26690	5–23,40,28,52,59	2,0,0,0

FGFTNEs	At2g04430	8–50,22,46,26,17,11,38,73	0,0,0,0,0,2,0
	At4g12720	8–48,22,47,26,17,11,38,73	0,0,0,0,0,2,0
	At5g47650	8–47,22,46,27,17,11,37,62	0,0,0,0,0,2,0
	At5g47240	6–60,22,47,43,48,75	0,0,0,0,0
	At2g04450	8–48,22,47,26,17,11,37,75	0,0,0,0,0,2,0

Coenzyme A pyrophosphatases	At5g45940	4–66,48,40,68	1,0,0
	At1g28960	4–111,48,40,74	1,0,0
	At2g33980	6–105,48,40,66,30,13	1,0,0,0,1

**Table 4:** The summary of specific duplicated pairs of Nudix genes identified by assessing the segmental duplication events unearthed in the study by Blanc et al. [41]. The size of the proteins, the percentage of sequence identity between the pair of proteins, the segment length (number of genes), the designated family of Nudix genes, and the rough estimation of the age of the duplication event are also presented. “CoAPs” designates coenzyme A pyrophosphatases, and “Ap_*n*_AHs” is representative of Ap_*n*_A hydrolases. In the study by Blanc et al. [41] the estimation of the age of the duplication event was conducted by the estimation of the synonymous substitution rates (Ks) between duplicated genes in sister regions. The duplications that are termed ‘recent’ contain median Ks values from 0.72 to 0.99 whereas those that are termed “old” have median Ks values from 1.82 to 6.03.

Gene 1	Length of protein	Gene 2	Length of protein	Sequence identity	Segment length	Family	Age
At1g18300	207	At1g73540	177	73%	285	DIPPs	Recent
At1g14860	176	At2g01670	182	78%	53	DIPPs	Recent
At1g12880	196	At3g26690	202	76%	26	DIPPs	Recent
At1g28960	273	At5g45940	222	50%	8	CoAPs	Old
At3g10620	216	At5g06340	227	51%	2	Ap_*n*_AHs	Old
At4g12720	282	At2g04430	283	54%	3	GFTNEs	Old
