# Supplementary material for: A Comprehensive Bioinformatics Analysis of the Nudix Superfamily in Arabidopsis thaliana
Source: Comp Funct Genomics. 2009 Jul 2;2009:820381. doi: 10.1155/2009/820381 (PMC2707057; doi:10.1155/2009/820381)
Supplement: Supplementary file 1 — Supplement Table A presenrs: Matrix of CLUSTALW scores from pairwise sequence alignment. Boxes where >30% sequence identity was detected by CLUSTALW alignment are colored in orange. Supplement Figure A presenrs: Sequence alignment of ApnA hydrolases from plants. The protein products of the Arabidopsis genes At1g30110, At3g10620 and At5g06340 and their counterparts from Lupinus angustifolius (gi:1888557), Oryza sativa (gi:50929793) and Hordeum vulgare (gi:2564253) are aligned using the CLUSTAL W algorithm. Motif 4 present only in the At1g30110 gene product is underlined in red. The N-terminal extensions forming putative preprotein sequences in all proteins excluding the At1g30110 gene product are underlined in green. [file 820381.f1.pdf]

Table A – Matrix of CLUSTALW scores from pairwise sequence alignment. Boxes where >30% sequence identity was detected by CLUSTALW alignment are colored in orange.

	At1g30110	At3g10620	At5g06340	At1g12880	At1g14860	At1g73540	At1g18300	At2g01670	At3g12600	At3g26690	At1g28960	At2g33980	At5g45940
At1g30110	N/A												
At3g10620	51	N/A											
At5g06340	48	51	N/A										
At1g12880	16	9	10	N/A									
At1g14860	17	13	12	47	N/A								
At1g73540	13	9	19	40	44	N/A							
At1g18300	14	7	8	38	47	73	N/A						
At2g01670	17	8	16	40	78	46	40	N/A					
At3g12600	7	5	15	44	42	38	36	45	N/A				
At3g26690	16	6	9	76	46	38	38	39	45	N/A			
At1g28960	17	8	6	10	15	10	12	5	8	6	N/A		
At2g33980	16	8	8	15	12	5	14	8	12	14	70	N/A	
At5g45940	11	6	14	11	10	18	11	10	10	7	50	48	N/A
At2g04430	13	6	4	7	10	13	9	7	9	10	5	10	7
At2g04450	15	13	10	15	17	22	15	7	10	11	8	12	8
At4g12720	13	10	7	9	15	12	12	7	12	8	4	4	4
At5g47240	14	16	9	8	7	16	10	10	11	9	8	13	8
At5g47650	15	12	13	7	11	6	10	8	11	4	4	6	12
At1g68760	18	12	14	9	17	12	10	17	17	10	12	21	14
At1g79690	16	7	8	11	15	6	9	9	8	4	7	10	15
At2g42070	12	10	9	7	16	9	11	9	12	5	5	11	6
At4g11980	12	13	12	6	10	5	6	9	18	10	10	9	8
At5g13570	16	13	7	16	10	15	16	7	11	12	8	12	10
At5g20070	18	8	9	10	12	16	17	8	9	8	5	4	11
At4g25440	9	16	5	7	12	7	1	10	6	8	3	5	6

	At2g04430	At2g04450	At4g12720	At5g47240	At5g47650	At1g68760	At1g79690	At2g42070	At4g11980	At5g13570	At5g20070	At4g25440
At1g30110												
At3g10620												
At5g06340												
At1g12880												
At1g14860												
At1g73540												
At1g18300												
At2g01670												
At3g12600												
At3g26690												
At1g28960												
At2g33980												
At5g45940												
At2g04430	N/A											
At2g04450	58	N/A										
At4g12720	54	61	N/A									
At5g47240	44	44	41	N/A								
At5g47650	44	47	48	48	N/A							
At1g68760	14	15	19	14	20	N/A						
At1g79690	15	14	8	8	13	15	N/A					
At2g42070	6	5	7	8	14	17	13	N/A				
At4g11980	10	9	9	11	13	13	6	3	N/A			
At5g13570	6	10	10	6	12	16	13	12	4	N/A		
At5g20070	7	13	7	11	10	18	8	18	5	9	N/A	
At4g25440	9	7	9	3	6	6	3	7	5	1	4	N/A

Figure A – Sequence alignment of Ap$_n$A hydrolases from plants

The protein products of the *Arabidopsis* genes At1g30110, At3g10620 and At5g06340 and their counterparts from *Lupinus angustifolius* (gi:1888557), *Oryza sativa* (gi:50929793) and *Hordeum vulgare* (gi:2564253) are aligned using the CLUSTAL W algorithm. Motif 4 present only in the At1g30110 gene product is underlined in red. The N-terminal extensions forming putative preprotein sequences in all proteins excluding the At1g30110 gene product are underlined in green.

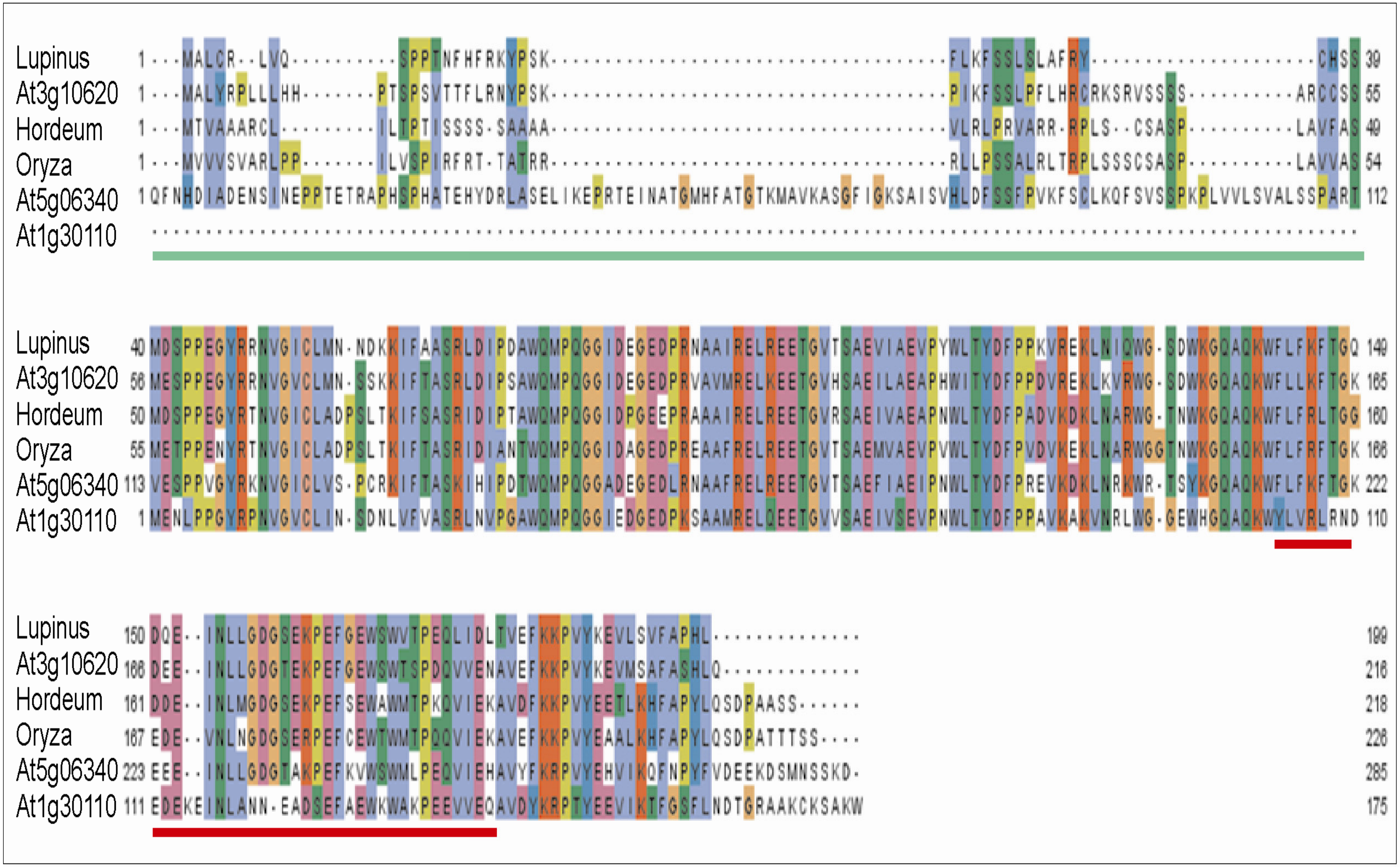
Lupinus
At3g10620
Hordeum
Oryza
At5g06340
At1g30110
